# An Initial In Vitro Investigation into the Potential Therapeutic Use of SupT1 Cells to Prevent AIDS in HIV-Seropositive Individuals

**DOI:** 10.1371/journal.pone.0037511

**Published:** 2012-05-23

**Authors:** Jonathan Fior

**Affiliations:** Section of Infectious Diseases and Immunopathology, Department of Clinical Sciences, Luigi Sacco Hospital, University of Milan, Milan, Italy; University of Pittsburgh Center for Vaccine Research, United States of America

## Abstract

HIV infection usually leads to a progressive decline in number and functionality of CD4+ T lymphocytes, resulting in AIDS development. In this study, I investigated the strategy of using inoculated SupT1 cells to move infection from HIV-1 X4 strains toward the inoculated cells, which should theoretically prevent infection and depletion of normal CD4+ T cells, preventing the development of AIDS-related pathologies. Interestingly, the persistent in vitro replication in SupT1 cells renders the virus less cytopathic and more sensitive to antibody-mediated neutralization, suggesting that replication of the virus in the inoculated SupT1 cells may have a vaccination effect in the long run. In order to mimic the scenario of a therapy in which SupT1 cells are inoculated in an HIV-seropositive patient, I used infected SupT1/PBMC cocultures and a series of control experiments. Infections were done with equal amounts of the wild type HIV-1 LAI virus. The SupT1 CD4+CD8+ T cell population was distinguished from the PBMC CD4+CD8− T cell population by FACS analysis. The results of this study show that the virus-mediated killing of primary CD4+ T cells in the SupT1/PBMC cocultures was significantly delayed, suggesting that the preferential infection of SupT1 cells can induce the virus to spare primary CD4+ T cells from infection and depletion. The preferential infection of SupT1 cells can be explained by the higher viral tropism for the SupT1 cell line. In conclusion, this study demonstrates that it's possible in an in vitro system to use SupT1 cells to prevent HIV infection of primary CD4+ T cells, suggesting that further exploration of the SupT1 cell line as a cell-based therapy against HIV-1 may prove worthwhile.

## Introduction

It was reported by a previous in vitro study that the X4 HIV-1 virus has a higher tropism for SupT1 cells than for primary CD4+ T cells [1]. Several hypotheses have been proposed as an explanation, most notably the higher surface expression of CD4 and CXCR4 receptors in SupT1 cells. It was also reported by HIV in vitro evolution studies that the persistent growth of the virus in the SupT1 cell line results in a less cytopathic virus with reduced capacity for syncytium formation, higher sensitivity to antibody-mediated neutralization, improved replication in SupT1 cells and impaired infection of primary CD4+ T cells [1]–[4]. The leukemic SupT1 cells are probably less susceptible to apoptosis than primary CD4+ T cells, allowing the lengthening of the period of virus production before the infected cell dies. This can improve viral replication efficiency and may also induce the virus to lose the costly escape mutations that hamper its replication ability; therefore, the selection of less virulent HIV-1 variants is the evolutionary route chosen by the virus. As reported by previous studies, it was observed increased viral production of HIV-infected CD4+T cells treated with caspase inhibitors that prevent cell death [5], [6], supporting the idea that viral replication efficiency improves in cells that are less susceptible to apoptosis. Another consideration regarding viral replication and viral evolution is related to the Vif protein. The HIV-1 accessory protein Vif is essential for replication in “nonpermissive” primary CD4+ T cells, in order to prevent hypermutation of newly-made HIV-DNA by cellular cytidine deaminase [7], [8]. Some “permissive” T cell lines (e.g., Jurkat and SupT1) lack deaminase activity [9] and fully support HIV-1 spread in the absence of Vif [10], [11], making Vif a nonessential viral protein for replication in SupT1 cells. The absence of cellular host restriction factors like cytidine deaminase may also explain the enhanced replication of the HIV-1 virus in the SupT1 cell line. Furthermore, the Vif protein shows that an essential protein for replication in primary CD4+ T cells might be a nonessential protein for replication in SupT1 cells. This supports the idea that many unnecessary genes could be deleted during the course of adaptation to growth in SupT1 cells, resulting in the development of less virulent HIV-1 variants. Considering the cited literature data, the SupT1 seems to be an interesting cell line to investigate as a possible cell-based therapy against HIV-1. HIV infection usually leads to a progressive decline in number and functionality of CD4+ T lymphocytes, resulting in AIDS development [12]. In this study, I investigated the strategy of using inoculated SupT1 cells to move infection from HIV-1 X4 strains toward the inoculated cells, which should theoretically prevent infection and depletion of normal CD4+ T cells, preventing the development of AIDS-related pathologies.

**Figure 1:**
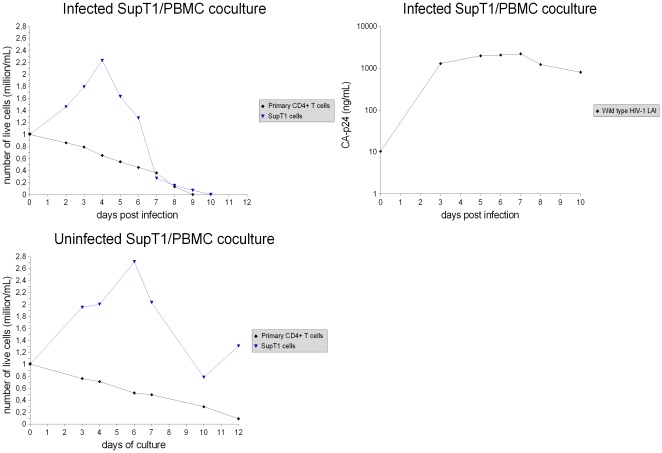
Infected SupT1/PBMC coculture and uninfected control. Infection was done with a virus input of 60 ng CA-p24 wild type HIV-1 LAI virus. In order to distinguish natural cell death from virus-mediated cell death (especially important for primary CD4+ T cells due to lower vitality and proliferation than SupT1 cells), the virus-mediated cell killing was determined by comparing the number of live cells in the infected coculture with the number of live cells in the uninfected control coculture. The results shown are from a representative experiment. The experiment was repeated ten times, and similar results were obtained (see Fig. 4 and Fig. 5 for details).

**Figure 2:**
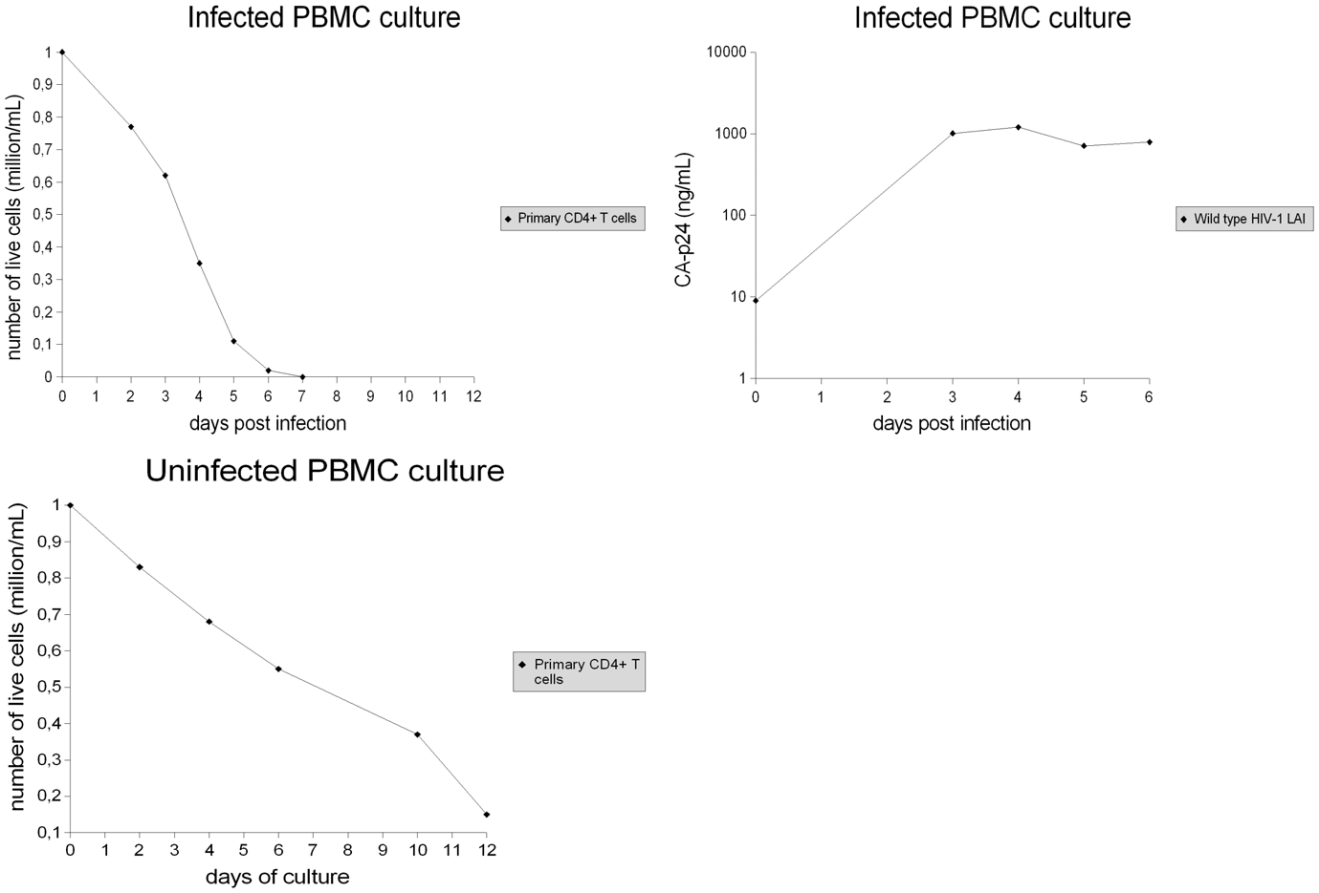
Infected PBMC culture and uninfected control. Infection was done with a virus input of 60 ng CA-p24 wild type HIV-1 LAI virus. In order to distinguish natural cell death from virus-mediated cell death, the virus-mediated cell killing was determined by comparing the number of live cells in the infected culture with the number of live cells in the uninfected control culture. The results shown are from a representative experiment. The experiment was repeated ten times, and similar results were obtained (see Fig. 4 and Fig. 5 for details).

**Figure 3:**
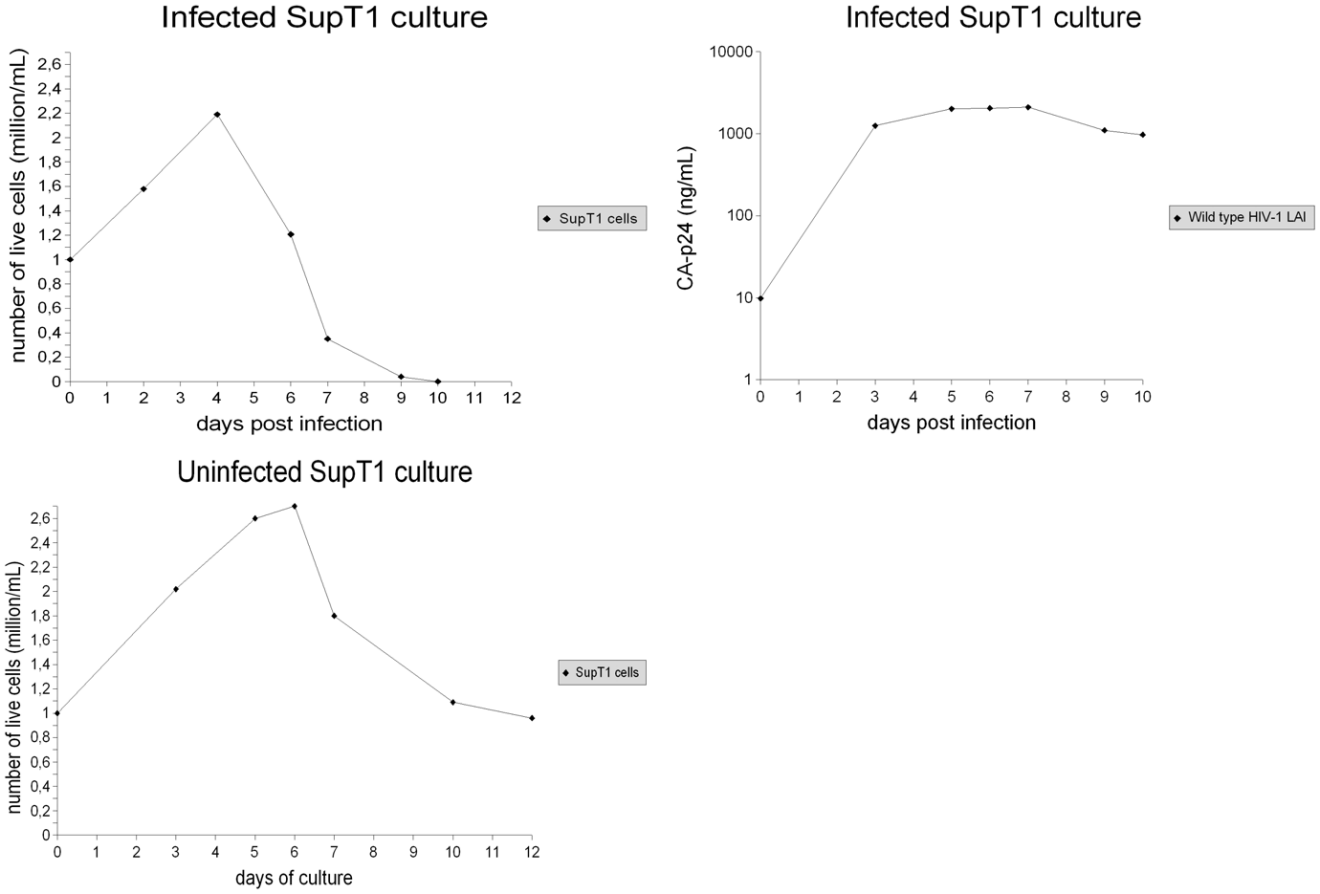
Infected SupT1 culture and uninfected control. Infection was done with a virus input of 60 ng CA-p24 wild type HIV-1 LAI virus. The virus-mediated cell killing was determined by comparing the number of live cells in the infected culture with the number of live cells in the uninfected control culture. The results shown are from a representative experiment. The experiment was repeated ten times, and similar results were obtained (see Fig. 4 and Fig. 5 for details).

**Figure 4:**
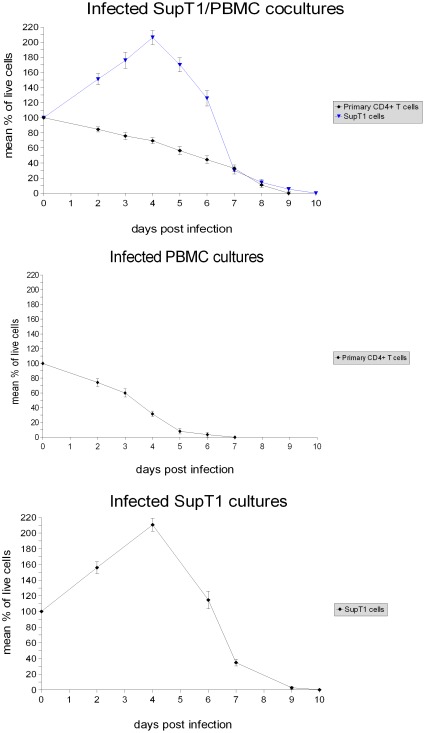
Data regarding repeatability of the experiments. All experiments in this study were repeated ten times; in each experiment, successive repetitions yielded consistent results. The collected data were summarized using means and standard deviations. This figure shows the data regarding the infected cultures. In the graphs, the lines show the mean values, and the error bars represent the standard deviations.

**Figure 5:**
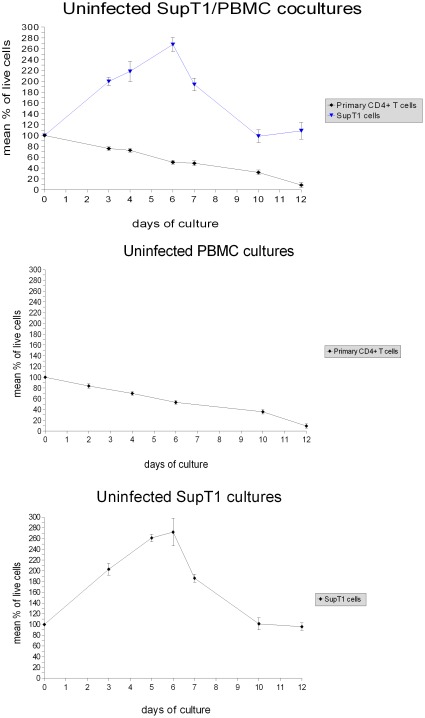
Data regarding repeatability of the experiments. All experiments in this study were repeated ten times; in each experiment, successive repetitions yielded consistent results. The collected data were summarized using means and standard deviations. This figure shows the data regarding the uninfected cultures. In the graphs, the lines show the mean values, and the error bars represent the standard deviations.

**Figure 6:**
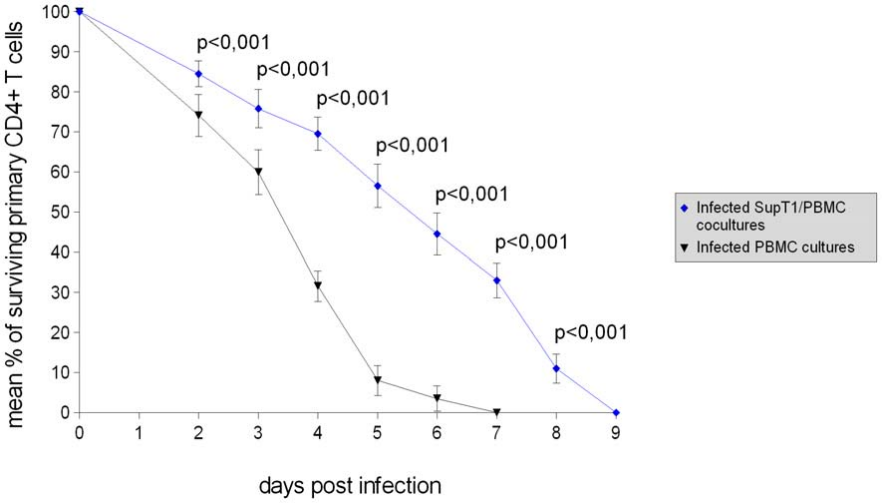
Examination of the difference in primary CD4+ T cell survival between the infected SupT1/PBMC cocultures and the infected PBMC cultures. In the graph, the lines show the mean values, and the error bars represent the standard deviations. Statistical significance (p) was assessed using the unpaired Student's t test.

Interestingly, the cited HIV in vitro evolution studies reported that the persistent in vitro replication in SupT1 cells renders the virus less cytopathic and more sensitive to antibody-mediated neutralization, suggesting that replication of the virus in the inoculated SupT1 cells may have a vaccination effect in the long run. In the real scenario of a therapy in which SupT1 cells are inoculated in an HIV-seropositive patient, the blood of the patient will contain a combination of SupT1 and normal CD4+T cells. In this situation the HIV-1 virus should preferentially infect SupT1 cells. In addition, the virus should completely eradicate SupT1 cells from the blood of the patient, preventing the uncontrolled replication of SupT1 cells. In order to mimic this scenario in my in vitro experiment, I used infected SupT1/PBMC cocultures and a series of control experiments. Infections were done with equal amounts of the wild type HIV-1 LAI virus. The SupT1 CD4+CD8+ T cell population was distinguished from the PBMC CD4+CD8− T cell population by FACS analysis. The wild type HIV-1 LAI virus replicated efficiently in all cultures; however, the virus-mediated killing of primary CD4+ T cells in the SupT1/PBMC cocultures was significantly delayed. This suggests that the preferential infection of SupT1 cells can induce the virus to spare primary CD4+ T cells from infection and depletion. The preferential infection of SupT1 cells can be explained by the higher viral tropism for the SupT1 cell line. Eradication of SupT1 cells was obtained on average 9–10 days after infection, and no evident allogeneic response against SupT1 cells was observed in the cocultures.

## Results

In agreement with the higher viral tropism for the “permissive” SupT1 cell line, in the SupT1/PBMC cocultures the HIV-1 virus preferentially infects and kills the leukemic SupT1 cell population. Therefore, 6 days after infection a large part of SupT1 cells were actively killed by the HIV-1 virus, while only a small fraction of primary CD4+ T cells were killed (Fig. 1).

By comparison, 6 days after infection all PBMC cultures showed complete eradication of primary CD4+ T cells (Fig. 2). These results suggest that the preferential infection of SupT1 cells can induce the virus to delay infection and depletion of primary cells.

### Examination of primary CD4+ T cell proliferation in the presence of SupT1 cells

In order to avoid misinterpretation of the data regarding primary CD4+ T cell survival, the proliferation of primary cells in the presence of SupT1 cells was examined in this study. Allogeneic immune response against SupT1 cells could be responsible for inducing activation and clonal expansions of primary CD4+ T cells. Therefore, the perceived survival of primary CD4+ T cells in the infected SupT1/PBMC cocultures could simply be a transient alloexpansion that eventually dies out. In such a case, the uninfected SupT1/PBMC cocultures would show higher proliferation of primary CD4+ T cells in comparison with the uninfected PBMC cultures. However, the growth curves of primary cells in these control experiments are similar (Fig. 1, Fig. 2), showing no evident sign of a strong alloexpansion. Another observation is that if the alloexpansion were present, it would be present in both infected and uninfected cocultures. Therefore, by comparing the number of live primary CD4+ T cells in the infected cocultures with the number of live primary CD4+ T cells in the corresponding uninfected control cocultures, even if there were the alloexpansion, the virus-mediated cell killing would still be shown. Considering this, if the preferential infection of SupT1 cells didn't prevent infection of primary CD4+ T cells, the killing of primary cells would be shown. However, in the first 5 days of infection no evident killing of primary CD4+ T cells is shown by comparing the infected SupT1/PBMC cocultures with the uninfected SupT1/PBMC cocultures (Fig. 1).

The killing of primary CD4+ T cells in the infected SupT1/PBMC cocultures started to be strong on average 7–8 days post infection, when the large majority of SupT1 cells were removed by the HIV-1 virus (Fig. 1). Thus, the presence of SupT1 cells seems to be directly responsible for the delayed virus-mediated killing of primary CD4+ T cells. In addition, no evident killing of SupT1 cells by allogeneic cytotoxic response is shown by comparing the number of live SupT1 cells in the uninfected SupT1/PBMC cocultures with the number of live SupT1 cells in the uninfected SupT1 cultures (Fig. 1, Fig. 3). This suggests that the HIV-1 virus was entirely responsible for killing and eradicating SupT1 cells in the infected cocultures. The results of all the experiments are summarized with means and standard deviations in Fig. 4 and Fig. 5, detailed examination of the difference in primary CD4+ T cell survival between the infected SupT1/PBMC cocultures and the infected PBMC cultures (with statistical significance) is provided in Fig. 6.

## Discussion

In this study, the killing of primary CD4+ T cells in the infected cocultures started to be vigorous when the large majority of SupT1 cells were removed by the HIV-1 virus. This suggests that in the real scenario of a cell-based therapy, after the complete eradication of the inoculated SupT1 cells by the HIV-1 virus, every potential therapeutic effect will end. Therefore, in order to maintain the supposed therapeutic effects, the inoculation with SupT1 cells will need to be repeated over time. Several serious safety issues need to be addressed before one can propose periodic inoculations with SupT1 cells as a therapy against the virally induced CD4+ T cell depletion. A first safety issue could be related to the risk of developing T-cell acute lymphocytic leukemia. However, as previously mentioned, the virus should completely eradicate SupT1 cells from the blood of the patient, preventing the uncontrolled replication of SupT1 cells. A second safety concern is that the cytokines produced by SupT1 cells may affect the immune system. In this regard, one may think about having IL-2, IL-12 or IL-15 expression plasmid transfected into SupT1 cells to enhance HIV-specific Th1 immune response [13], but the risk of introducing an uncontrolled cytokine loop may hamper this approach.

Another issue could be related to the development of a strong allogeneic response against SupT1 cells. Although no killing of SupT1 cells by allogeneic response was observed in my experimental setting, it is possible that a patient could develop a specific immune response against SupT1 cells over time. Strong killing of SupT1 cells by a specific immune response could limit the supposed therapeutic effects of the inoculation to a very short time. However, considering that the blood of a patient with T-cell leukemia contains a combination of normal and leukemic T cells, one could argue that if the immune system were able to efficiently eradicate leukemic T cells, then T-cell leukemia would not be such an aggressive disease. In this regard, it should be mentioned that an HIV-1 variant that selectively replicates in leukemic T cells was proposed as a therapeutic virus against CXCR4-expressing T-ALL malignancies [14]. Finally, it should also be mentioned that human inoculation with in vitro grown T cells was already performed in T cell vaccination [15], [16] and Adoptive T cell therapy [17], [18]. Concerning T cell vaccination, the T cell line cells are weakened with radiation to make them unable to replicate, these cells are then inoculated in the patient. In order to make the inoculation with SupT1 cells safer, a similar protocol could be proposed. In conclusion, this study demonstrates that it's possible in an in vitro system to use SupT1 cells to prevent HIV infection of primary CD4+ T cells, suggesting that further exploration of the SupT1 cell line as a cell-based therapy against HIV-1 may prove worthwhile.

## Materials and Methods

### Cells

The C33A cervical cancer cell line was obtained from the American Type Culture Collection, ATCC HTB31 [19]. C33A cells were cultured in Dulbecco's modified Eagle's medium supplemented with 10% FBS, 1% penicillin/streptomycin at 37°C and in 5% CO_2_.

The SupT1 lymphoblastoid CD4+ T cell line was obtained from the American Type Culture Collection, ATCC CRL-1942 [20]. SupT1 cells were cultured in RPMI 1640 supplemented with 10% FBS, 1% glutamine, 1% penicillin/streptomycin at 37°C and in 5% CO_2_.

Freshly isolated PBMC obtained from buffy coats of healthy HIV-seronegative donors (Blood Transfusion Center, L. Sacco Hospital) were cultured in RPMI 1640 supplemented with 10% FBS, 1% glutamine, 1% penicillin/streptomycin, 100 units/mL IL-2 and 5 micrograms/mL phytohemagglutinin at 37°C and in 5% CO_2_. PBMC from different donors were cultured separately and never mixed in order to avoid allogeneic response. The protocols used in this study were approved by the L. Sacco Hospital Ethics Committee, and the blood samples used in this study were obtained from donors who gave written informed consent.

#### SupT1/PBMC coculture

48 h after activation with PHA, PBMC containing 5 million primary CD4+ T cells were washed with medium and mixed with 5 million SupT1 cells, and the cell mixture was cultured in 5 mL of complete medium.

#### PBMC culture

48 h after activation with PHA, PBMC containing 5 million primary CD4+ T cells were resuspended and cultured in 5 mL of complete medium.

#### SupT1 culture

5 million SupT1 cells were resuspended and cultured in 5 mL of complete medium.

### Infections

Virus stocks were generated by transfection of C33A cells. All infections were done with a virus input of 60 ng CA-p24 wild type HIV-1 LAI virus, and samples were taken from the cultures for anti-CD4/CD8 FACS analysis and CA-p24 ELISA analysis.

### Flow cytometry

The number of live cells in each culture was determined by FACS analysis. The PBMC CD4+CD8− T cell population was distinguished from the SupT1 CD4+CD8+ T cell population by FACS analysis using DAKO Monoclonal Mouse Anti-Human CD4, Clone MT310, conjugated with R-phycoerythrin (RPE), and DAKO Monoclonal Mouse Anti-Human CD8, Clone DK25, conjugated with fluorescein isothiocyanate isomer 1 (FITC). Gates for PBMC (CD4+CD8−) and SupT1 (CD4+CD8+) were set with a separate control culture. After staining, the cells were analyzed in a FACS Calibur system running with CellQuest Pro software (BD Biosciences).

### ELISA

The CA-p24 levels in the culture supernatants were measured by ELISA as described by the manufacturer (Abbott Laboratories).

### Statistical analysis

This study was repeated with ten different PBMC samples, and similar results were obtained. The collected data were summarized using means and standard deviations. Statistical significance (p) was assessed using the unpaired Student's t test.
